# Editorial: Translational advances in Alzheimer's, Parkinson's, and other dementia: molecular mechanisms, biomarkers, diagnosis, and therapies, volume III

**DOI:** 10.3389/fnagi.2023.1352988

**Published:** 2024-01-08

**Authors:** Jiehui Jiang, Kuangyu Shi, Kenneth S. Hettie, Chih-Yu Hsu, Woon-Man Kung

**Affiliations:** ^1^School of Life Science, Institute of Biomedical Engineering, Shanghai University, Shanghai, China; ^2^Department of Nuclear Medicine, Inselspital, Bern University Hospital, University of Bern, Bern, Switzerland; ^3^Department of Informatics, Technical University of Munich, Munich, Germany; ^4^Department of Radiology, Molecular Imaging Program at Stanford (MIPS), Stanford University School of Medicine, Stanford, CA, United States; ^5^Department of Otolaryngology - Head and Neck Surgery, Molecular Imaging Program at Stanford (MIPS), Stanford University School of Medicine, Stanford, CA, United States; ^6^School of Transportation, Fujian University of Technology, Fuzhou, China; ^7^Division of Neurosurgery, Department of Surgery, Taipei Tzu Chi Hospital, Buddhist Tzu Chi Medical Foundation, New Taipei City, Taiwan; ^8^Department of Exercise and Health Promotion, College of Kinesiology and Health, Chinese Culture University, Taipei, Taiwan

**Keywords:** Alzheimer's disease, Parkinson's disease, dementia, neurodegenerative disorders, big data mining, imaging methods, bioinformatic applications

This Research Topic (RT) is a series continuation of two previous prominent RTs, Volume I (Jiang et al., 2022b) and Volume II, (Jiang et al., 2022a) published in 2022. These previous publications reviewed 69 and 57 articles, respectively. Volume III is the latest collection on the topic and comprises 41 globally sourced articles, contributing significant updates from diverse viewpoints on this important theme between 2022 and 2023. In total, 362 authors, including researcher scientists and clinicians, who were actively engaged in the field, contributed to these Research Topics.

The ability to detect neurodegenerative dementia at an early stage continues to receive increased attention. Common etiologies include Alzheimer's disease (AD), Parkinson's disease (PD), and vascular dementia (VD) (Andjelkovic et al., 2023; Andrews et al., 2023; Louis et al., 2023). Study results have shown that changes in the underlying molecular biology of such cognitive impairments are associated with dementia. Recently, research studies have demonstrated that molecular imaging techniques can capture small alternations in patients with dementia. Improvements in biotechnologies, such as primarily positron emission tomography (PET) and magnetic resonance imaging (MRI), continue to demonstrate molecular imaging techniques that capture the small alterations and events in patients with dementia (Borghammer, 2021; Roda et al., 2022). Moreover, artificial intelligence is emerging as a critical tool in bioinformatics research (Mei et al., 2021; Goyal et al., 2022). However, detection of AD and other dementias in their “early phase” remains a challenge in the current standard of care.

To address current challenges, the articles in this Volume are categorized into sections based on their underlying disciplinary approach, which seeks to identify and/or treat dementia.

## Genetics

Luo et al. identified REPS1 as the hub gene for AD and VD based on 3 datasets. Qiu et al. identified a novel rare variant c. - 2067A > C and found that it may reduce Amyloid-β (Aβ) deposition in the hippocampus. Wang et al. implemented whole-exome sequencing (WES) and whole-genome sequencing (WGS) to mine data from 3,959 PD patients and 2,931 healthy controls. The results showed that COL6A3 and TH were associated with PD. Dawson et al. discovered that dysregulated expression of ancient retrovirus insertions in the human genome are present in AD. Ma et al. developed and utilized predictive models to assess the risk of developing the disulfidptosis subtype in patients with AD, finding that disulfidptosis is strongly associated with AD.

## Biomarkers: molecular

Leirós et al. reported that higher serum levels of vitamin A, vitamin D, transthyretin (TTR), albumin, selenium (Se), and uric acid (UA) could act as protective factors against cognitive decline based on multiple logistic regression, whereas higher body mass index (BMI) could act as a risk factor. Chen Y.-T. et al. combined plasma α-synuclein level and Motor Dysfunction Questionnaire (MDQ) to discriminate between dementia with Lewy bodies (DLB) and AD. Wu et al. used an engineered tau fragment 4RCF as a substrate to investigate misfolded tau from diseased AD and other tauopathy brains and were able to seed the recombinant 4RCF substrate. Their conclusion from the data were that it may be useful as a biomarker for the diagnosis of AD and related tau lesions. In a study performed by Nardini et al., utilizing the mechanical characteristics of red blood cells, they used a standard linear solid model that was found to demonstrate excellent classifications for determining AD. Lu et al. used different mouse models to find that similar pathogenic features in aging, AD, and perioperative neurocognitive disorder (PND) from dysregulation of protein kinase C (PKC)/protein kinase (PKR) activity may be related to its pathogenesis. Based on the Parkinson's Progression Markers Initiative (PPMI) database, Sheng et al. found that the concentration and changes of Neurofilament light chain protein (NfL) in cerebrospinal fluid (CSF) could be used to identify the cognitive progress in PD patients.

Chen D. et al. surveyed significant changes in the vitamin profile in multiple system atrophy (MSA). The results from receiver operating characteristic (ROC) curves suggested that vitamin A, folate, and vitamin C could discriminate between MSA and normal controls. Yang et al. applied high-resolution mass spectrometry to detect metabolites in AD plasma samples, wherein they found significant fluctuations in the levels of plasma metabolites, PAGln, and L-arginine in the AD group. Using 6 datasets derived from AD frontal cortex samples, Jin et al. employed a combination of bioinformatics analysis and machine learning (ML) to screen out thioredoxin interaction protein (TXNIP), early growth Response 1 (EGR1), and insulin-like growth factor binding protein 5 (IGFBP5), as diagnostic markers for AD. He et al. evaluated the gut microbiota-brain-cognition interaction, wherein their findings suggested that there is an intrinsic association between gut microbiota changes, brain atrophy, and cognitive decline. Collins et al. determined a positive association between plasma NfL levels measured by single molecule array technology and cognitive decline with aging. They suggested that although NfL level was associated with cognitive decline, it did not mediate the relationship between age and cognition.

## Biomarkers: imaging

Tsui et al. quantified the comprehensive morphological characteristics of Aβ plaque deposition in the brain using an AD mouse model. In using ^11^C-CFT coupled with PET imaging, Chen M.-J. et al. determined that there is a significant contribution to the disease severity of progressive supranuclear palsy (PSP) by the striatal dopamine transporter (DAT) bindings (caudate). By utilizing the functional covariance connection strength method, Du et al. indicated that characteristics of olfactory-related brain networks can potentially be used as neuroimaging biomarkers for characterizing PD states.

Li S. et al. performed ^131^I-metaiodobenzylguanidine (MIBG) scintillation imaging on 37 subjects, wherein they determined that MIBG uptake rate could assist in PD diagnosis. Hao et al. combined retinal imaging and multimodal MRI to show promising results in identifying AD patients. Bao et al. assessed that regional homogeneity (ReHo) and functional connectivity (FC) abnormalities may occur in the basal ganglia, limbic regions, and cognitive control cortex during the early stage of gait freezing. Isernia et al. found that depression fully mediates a relationship between frailty and thickness of the superior limbic and rostral medial frontal gyrus cortex.

## Computer-aided diagnosis

Based on the diagnostic data of a questionnaire survey, Zhao et al. discerned the bagging method model to have the best diagnostic effect, whereas principal component analysis (PCA) has the best feature selection effect, both after having compared various models and feature selection algorithms. Deng et al. used nomogram and the XGBoost ML method to establish an effective and relatively accurate prediction model (accuracy of 71.86%) of excessive daytime sleepiness (EDS) in PD.

## Therapies

Khachatryan et al. focused on the inclusion of a cognitive task during the Gamma ENtrainment Using Sensory stimulation (GENUS) session. Their results proved that the inclusion had a positive effect on the strength and extent of the gamma entrainment, followed by an improvement in the therapeutic effect of GENUS (Khachatryan et al.). Zhou Z. et al. performed cluster analysis in a cohort of 1,217 individuals with early-onset PD and identified 3 clinical subtypes (mild, intermediate, and severe). Wan et al. specified that Baduanjin exercise was effective in improving cognition. Kainuma et al. reported that the increase of plasma metabolite levels after 6 months of hachimijiogan (HJG) administration was significantly higher than that of the control group.

Mei et al. investigated the effects of bright light therapy (BLT) on AD patients and caregivers. Their study showed that there was an inhibitory effect of BLT on depression. Lin et al. proposed a prospective, observational cohort study to determine the effects of BLT on migraine patients with sleep disorders.

## Reviews

Several reviews are included in this section of the Research Topic. Huang et al. reviewed the work related to PD and subjective cognitive decline (SCD) and they found that PD with SCD occurred with metabolic changes to the brain. Regmi et al. discussed the strategies and limitations of using mesenchymal stem/stromal cells (MSCs) therapy for AD. Subramanian et al. described the different mechanisms and signaling regulations, highlighting the trilateral association of autophagy, the mammalian target of the rapamycin (mTOR) pathway, and AD with a description of inhibiting drugs/molecules of mTOR, a strategic target in AD. Peng et al. investigated bottlenecks in utilizing therapeutic strategies toward AD, wherein they focused on anti-Aβ, anti-tau drugs, and mitochondrial targeted therapy to suggest future directions for AD drug therapies.

Li G. et al. conducted a network meta-analysis that aimed to evaluate the efficacy of acupuncture in animal models of VD, which provided evidence that acupuncture significantly improved cognitive function in rats with VD. Another meta-analysis was conducted by Song et al. to show the effects of D-ribose on cognition in rodents. They summarized the outcomes of eight trials involving 289 rodents and conducted a systematic review. The results of their study identified D-ribose therapy as a cause of cognitive impairment with cognitive ability worsening with increasing dose. Bian et al. conducted a systematic review and meta-analysis to estimate the diagnostic value of image-based ML for PD. The findings from their paper suggested that image-based ML can be used as a potential tool for PD diagnosis. As synaptic plasticity disorders play key roles in AD, Zhang et al. implemented a bibliometric analysis to gain insights into how systematic synaptic plasticity affects the progress of AD.

## Miscellaneous

Chen T.-Y. et al. conducted a retrospective analysis and rated white matter hyperintensities (WMHs) visual scores on 449 patients with DLB. This study obtained useful information that WMHs were associated with cognitive impairment (Chen T.-Y. et al.). Zhou X. et al. conducted a longitudinal study that enrolled 2,100 PD subjects and demonstrated that fatigue may be a risk factor for increased disease severity.

## Conclusions

A number of articles in this Research Topic collection focus on the application of bioinformatics and computational biology, with some studies discussing disease susceptibility genes. In addition, several researchers used molecular methods, novel imaging techniques, and imaging analysis to explore biomarkers. Even though other novel clinical treatments have not been covered here, the multidisciplinary perspectives of the studies included in the Research Topic facilitate and improve understanding of such disease mechanisms, helping to identify novel potential biomarkers and treatment targets.

## Author contributions

JJ: Writing—original draft. KS: Writing—original draft. KH: Writing—review & editing. C-YH: Writing—review & editing. W-MK: Writing—review & editing.
